# Challenges in Identifying and Diagnosing Asbestos-Related Diseases in Emerging Economies: A Global Health Perspective

**DOI:** 10.5334/aogh.4871

**Published:** 2025-09-18

**Authors:** Priyanka Roy, Ankita Raheja, Khushi Prajapati, Shubhajeet Roy, Mainak Bardhan, Arthur L. Frank

**Affiliations:** 1 Directorate of Factories, Government of West Bengal, 1, K.S. Roy Road, Kolkata-1, West Bengal, India; 2 Hbt Medical College And Dr R N Cooper, Municipal General Hospital, Mumbai, Maharashtra, India; 3 Faculty of Medical Sciences, King George’s Medical University, Lucknow, India; 4 The Dr. John T. Macdonald Foundation Department of Human Genetics, University of Miami Miller School of Medicine, Miami, FL, USA; 5 The John P. Hussman Institute for Human Genomics, University of Miami Miller School of Medicine, Miami, FL, USA; 6 Clinical Professor, Dornsife School of Public Health, Drexel University, Philadelphia, PA, USA

**Keywords:** asbestos, asbestosis, mesothelioma, diagnosis, developing countries, occupational health, imaging, global burden

## Abstract

*Background:* Asbestos, a durable fibrous silicate once widely used for its thermal resistance, remains in use in countries like India and China despite being banned in over 70 nations and classified as a Group 1 carcinogen by IARC. Prolonged occupational exposure causes asbestosis, lung cancer, and malignant pleural mesothelioma, but in Low and Middle-Income Countries (LMICs) the true burden is underreported due to weak regulation, low awareness, limited diagnostics, and inadequate occupational health systems.

*Objectives:* This review aimed to examine the epidemiological patterns and diagnostic challenges of Asbestos-Related Disease (ARDs) in emerging economies, with a focus on the applicability and limitations of existing and emerging diagnostic strategies.

*Methods:* We conducted a narrative review of peer-reviewed literature, global databases (WHO, IARC), and recent cohort and cross-sectional studies, sourcing articles through structured keyword searches in PubMed, Scopus, and Google Scholar. Diagnostic approaches were compared across diverse healthcare settings, emphasizing radiological, histopathological, and functional tools. The review also assessed the utility of newer technologies, including low-dose CT (LDCT), ultra-low-dose CT (ULDCT), magnetic resonance imaging (MRI), FDG-PET is Fluorodeoxyglucose Positron Emission Tomography (FDG-PET), breath biomarkers using gas chromatography-mass spectrometry (GC-MS), and digital tomosynthesis (DTS).

*Findings:* LDCT and ULDCT showed superior sensitivity for early detection of pleural abnormalities like circumscribed pleural plaques and diffuse thickening, yet distinguishing benign from malignant lesions remains difficult without biopsy. Diffusion capacity of the lungs for carbon monoxide (DLCO) emerged as a sensitive but nonspecific pulmonary function marker. Histopathological confirmation of mesothelioma remains the gold standard but is rarely accessible in low-resource settings.

*Conclusion:* Addressing the diagnostic gap in ARDs in LMICs requires systemic strengthening of occupational health surveillance, better regulatory enforcement, expanded access to advanced diagnostic tools, and targeted clinician training. Without urgent intervention, the burden of asbestos exposure will remain an escalating public health crisis.

## Introduction

Asbestos is a group of six naturally occurring crystalline hydrated silicate minerals characterized by their fibrous structure, with an aspect ratio (length to diameter) exceeding 3:1 [1]. It includes a serpentine form chrysotile (white asbestos) and five amphiboles, namely crocidolite (blue asbestos), amosite (brown asbestos), anthophyllite, tremolite, and actinolite [2]. These fibers can vary significantly in size, with lengths extending up to 500 microns and diameters ranging from 0.5 to 50 microns based on their chemistry and fiber morphology [1]. Because of its tensile strength, heat resistance, and durability, asbestos has been incorporated into thousands of industrial and consumer products, ranging from construction materials to automotive parts. Since no form of asbestos is safe, prolonged exposure to asbestos fibers poses severe health risks, including asbestosis, lung cancer, and mesothelioma, underscoring the need for strict occupational safety measures and regulatory control [3, 4].

Asbestos has been commercially mined and used in North America since the mid-1800s; its use increased significantly during World War II. A full ban was enacted in 1989, but the court system reversed the ban in 1991 [5]. The United States Environmental Protection Agency (EPA) banned all new uses of asbestos, although uses developed before 1989 were still permitted. More recently, the use of chrysotile for specific uses in factories was banned, but this ban is being challenged in court. Although it has been banned in more than 70 countries, a lot of developing countries still use asbestos, where it remains a low-cost material for construction and manufacturing [1, 4]. For example, between 2000 and 2004, asbestos consumption rose by 20% in Indonesia, 31% in India, 48% in China, and 51% in Thailand [6]. Based on a 2016 estimate of the world’s asbestos consumption, India, a developing nation, now consumes the most asbestos (308,000 metric tons) [7]. Despite international conventions such as the International Labour Organization’s Asbestos Convention (C162) and the Basel Convention, global consensus on a full ban remains elusive, with some countries blocking asbestos’ inclusion in the Rotterdam Convention [8–10]. The ongoing use of asbestos poses a major global health threat. The World Health Organization (WHO) estimates that occupational exposure causes over 250,000 deaths annually, accounting for more than 70% of all work-related cancer deaths worldwide [11]. These figures translate into more than 1.5 million disability-adjusted life years (DALYs) lost each year [12]. Chrysotile has been linked to pulmonary fibrosis and mesothelioma in humans, even at low levels of exposure, necessitating the application of the precautionary principle globally to phase out its use [13, 14].

**Figure 1:**
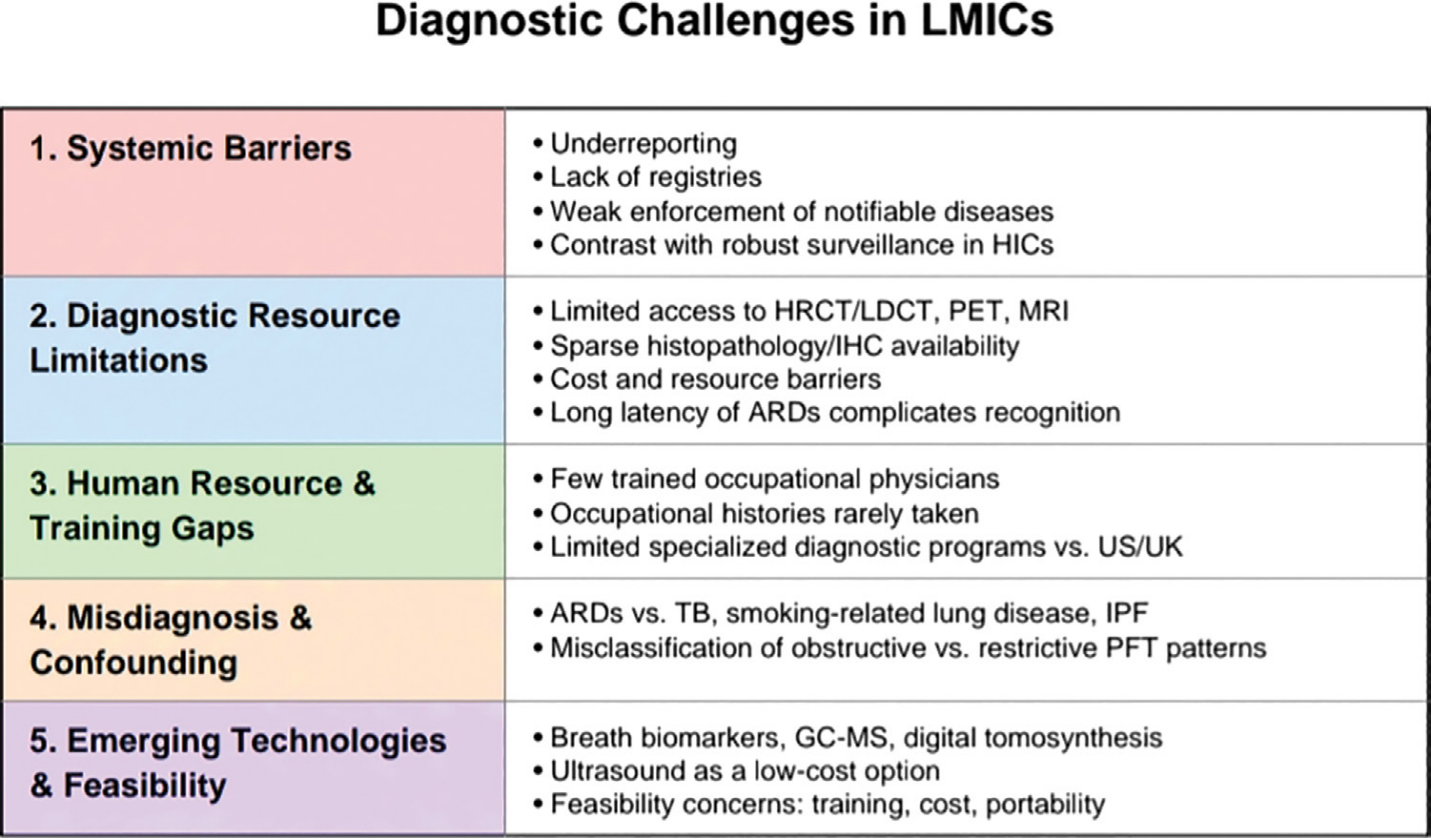
Five pillars of diagnostic challenges in LMICs.

Asbestos has been classified as a known human carcinogen by the United States Department of Health and Human Services (HHS), the EPA, and the International Agency for Research on Cancer (IARC) [2, 8]. Heavy exposure to asbestos causes asbestosis, while even low exposure to asbestos increases the risk of benign pleural diseases, especially pleural plaques [14]. Asbestos-related lung diseases comprise pleural and parenchymal diseases that may be benign or malignant. Non-malignant diseases include pleural plaques, rounded atelectasis, benign asbestos-related pleural effusion (BAPE), diffuse pleural/ and or peritoneal thickening (DPT), and asbestosis. Malignant diseases related to asbestos exposure include malignant pleural mesothelioma and primary lung cancer, and other cancers, e.g., GI and kidney [13] (Table 1). Importantly, the health effects of asbestos are already well documented in the literature. The unique focus of this review is therefore not to revisit disease mechanisms, but rather to highlight the diagnostic challenges specific to low- and middle-income countries (LMICs). These include systemic underreporting, limited access to advanced imaging and histopathology, a lack of clinician expertise in occupational health, and limited expertise impeding disease recognition (Figure 1). Addressing these diagnostic gaps is critical to accurately assessing disease burden, improving patient outcomes, and guiding regulatory action in countries where asbestos use continues to rise. While developed nations have established robust surveillance systems and diagnostic pathways, LMICs face the dual challenge of rising asbestos use and weak occupational health infrastructures. The following sections, therefore, focus on these diagnostic barriers, examining both the systemic and technical obstacles that hinder recognition of asbestos-related diseases (ARDs) in LMICs, and evaluating emerging tools that may offer more feasible solutions in resource-limited environments.

**Table 1:** Brief overview of asbestos-related diseases (ARDs).

DISEASE	TYPE	KEY FEATURES	DIAGNOSTIC NOTES / CHALLENGES
Pleural plaques	Non-malignant	Localized, often calcified pleural thickening; marker of exposure	Usually asymptomatic; easily identified on CT; not a risk factor by itself for mesothelioma but indicates exposure history
Benign asbestos-related pleural effusion (BAPE)	Non-malignant	Small, unilateral or bilateral pleural effusions	Often self-resolving; may mimic infection or malignancy; requires exclusion of cancer
Diffuse pleural thickening (DPT)	Non-malignant	Extensive pleural fibrosis; may restrict lung expansion	Can be misclassified as other pleural pathology; reduced specificity in plain radiographs
Asbestosis	Non-malignant parenchymal fibrosis	Progressive pulmonary fibrosis with restrictive physiology	Radiologic features overlap with idiopathic pulmonary fibrosis; careful exposure history essential
Lung cancer	Malignant	Elevated risk in exposed workers, synergistic with smoking	Diagnosis follows standard oncologic pathways, but exposure attribution often overlooked in LMICs
Malignant pleural mesothelioma (MPM)	Malignant	Aggressive cancer of pleura, long latency	Histopathology with immunohistochemistry required for confirmation; diagnosis often delayed in LMICs due to lack of access to pathology

## Epidemiology

ARDs—including mesothelioma, asbestosis, and lung cancer—remain a major contributor to global occupational mortality. Mesothelioma, asbestosis, and lung cancer from occupational exposures caused 250,000 yearly deaths and at least 1,523,000 DALYs in 2004 worldwide [15] (Table 2). It is noteworthy that there is a substantial difference in the incidence of mesothelioma. Despite declining asbestos use in many high-income countries, the global burden persists and is increasingly concentrated in LMICs, where asbestos consumption remains high and occupational health systems are weak.

**Table 2:** Mesothelioma incidence in 2020 by region (ASR per 100,000 persons).

REGION / COUNTRY GROUP	WHO INCOME GROUP	AGE-STANDARDIZED RATE (ASR)	INTERPRETATION
Northern Europe	High-income	1.4	Reflects strong surveillance and diagnostic capacity
Australia & New Zealand	High-income	1.3	Comparable to Europe; robust reporting systems
Western Europe	High-income	0.79	Consistent with historic asbestos use and effective case identification
Southern Europe	High-income	0.70	Underlines strong reporting despite declining asbestos use
Southern Africa	Upper-middle	0.55	Intermediate rate; may reflect partial underreporting
South-Central Asia	Low-/middle-income	0.10	Artificially low; reflects diagnostic gaps, not absence of exposure
Middle Africa	Low-income	0.07	Very likely underestimation due to lack of surveillance
Western Africa	Low-income	0.06	Minimal reporting; cases likely unrecognized
Eastern Africa	Low-income	0.06	Surveillance systems weak or absent
Caribbean	Upper-middle	0.05	Likely underreporting despite asbestos exposure risks

A striking feature of ARD epidemiology is the disparity between developed and developing nations due to difficulty in diagnosis and significant underreporting (Figure 1). Age-standardized rates (ASR) of mesothelioma in 2020 were 1.4 per 100,000 in Northern Europe and 1.3 per 100,000 in Australia/New Zealand, compared with 0.10 in South-Central Asia and 0.06 in parts of Africa. Data from 2020 clearly show that, in comparison to regions in Southern Africa and South-Central Asia, ASR in Northern Europe, Australia, and New Zealand are significantly higher [16]. These differences do not reflect true variations in exposure, but rather the impact of diagnostic undercapacity and underreporting. In high-income countries, access to advanced imaging, histopathology, and immunohistochemical panels allows for reliable case confirmation. For instance, the appropriate use of a panel of immunohistochemical markers is necessary for the confirmation of a mesothelioma diagnosis; however, this option is frequently unavailable in low-income countries. Therefore, there appears to be a lack of reporting of mesothelioma cases in developing nations [17].

The consequence is a systematic underestimation of ARDs in regions with some of the highest ongoing asbestos use, such as India, China, and parts of Southeast Asia. Workers and communities remain exposed through occupational handling, informal industries, and deteriorating asbestos-containing buildings, yet the epidemiological data fail to capture the true scale of disease. This underdiagnosis not only masks the public health crisis but also hampers the ability of governments to regulate asbestos, allocate resources, or compensate affected workers. Inhaling fibers from contaminated air in the workplace, ambient air near point sources, or indoor air in homes and buildings with friable asbestos materials are the main ways people become exposed to asbestos. In addition, asbestos exposure occurs when products containing asbestos are installed or used or removed in workplaces and homes. Many buildings still contain friable materials that contain amphiboles and chrysotile, which can lead to exposure to these materials during maintenance, alteration, removal, and demolition; exposure can also happen when buildings are damaged by natural disasters [6, 8].

Asbestos can enter the body through inhalation, with fine dust settling in the alveoli and by ingestion. These insoluble fibers can migrate within and from the lungs, leading to severe health consequences in the lungs and surrounding pleura. Fibers can also migrate to many other organs. According to the WHO, any inhaled asbestos dust can cause mesothelioma of the pleura or peritoneum, bronchial carcinoma, ovarian carcinoma, and pulmonary fibrosis, potentially resulting in respiratory failure and death. Additionally, smoking combined with asbestos exposure significantly increases the risk of developing bronchial cancer [18, 19].

## Challenges of Diagnosis

Although the spectrum of ARDs is well-characterized in the medical literature, translating this knowledge into timely and accurate diagnoses remains a profound challenge in many parts of the world. High-income countries benefit from decades of occupational health surveillance, established cancer registries, and access to advanced diagnostic technologies, which together enable systematic case detection and monitoring. By contrast, in LMICs, the diagnostic process is undermined at multiple levels—ranging from weak surveillance and underreporting, to shortages of imaging and pathology infrastructure, to limited clinical expertise in occupational medicine. These gaps result in a paradoxical situation: countries with the highest current asbestos consumption often report the lowest incidence of ARDs, not because of true absence, but because of barriers to recognition and confirmation.

The following subsections examine these diagnostic obstacles in greater detail, beginning with systemic barriers related to underreporting and poor surveillance, and then turning to technical limitations in diagnostic tools and workforce capacity (Figure 1).

**Figure 2:**
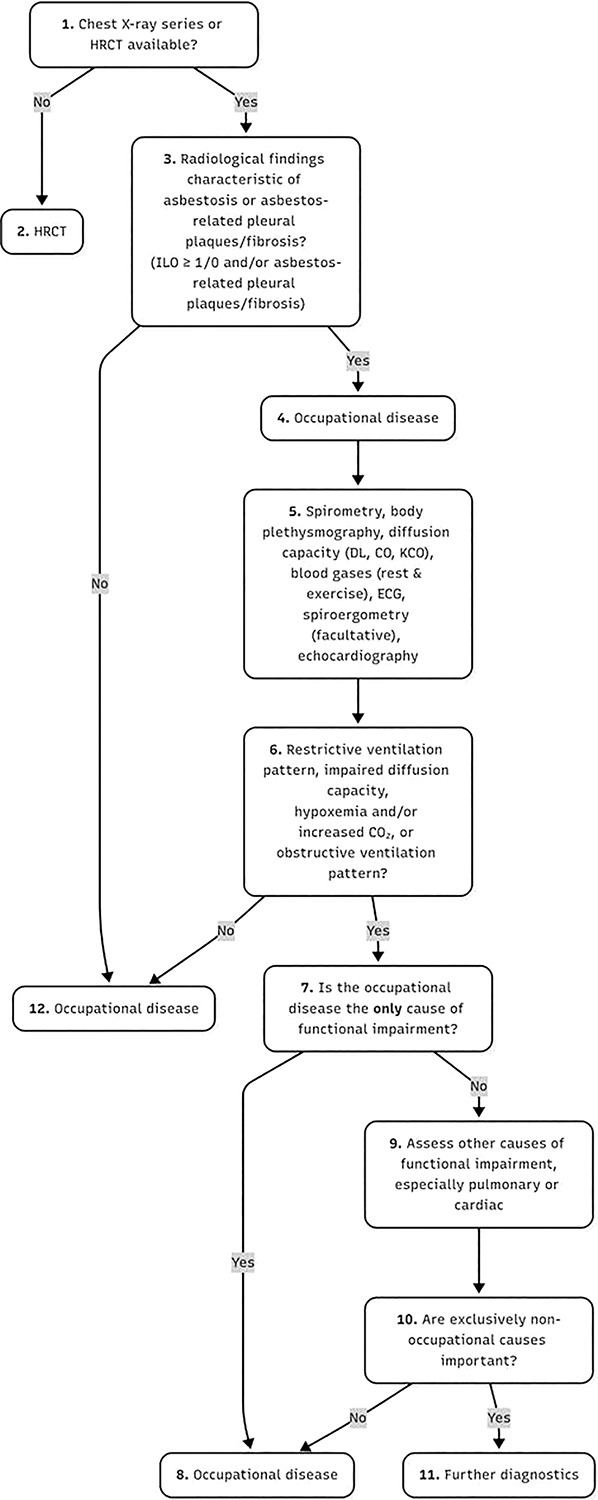
The algorithm of medical expert examination for the diagnosis of asbestosis or asbestos‐related pleural plaques/fibrosis according to the German guidelines.

**Figure 3:**
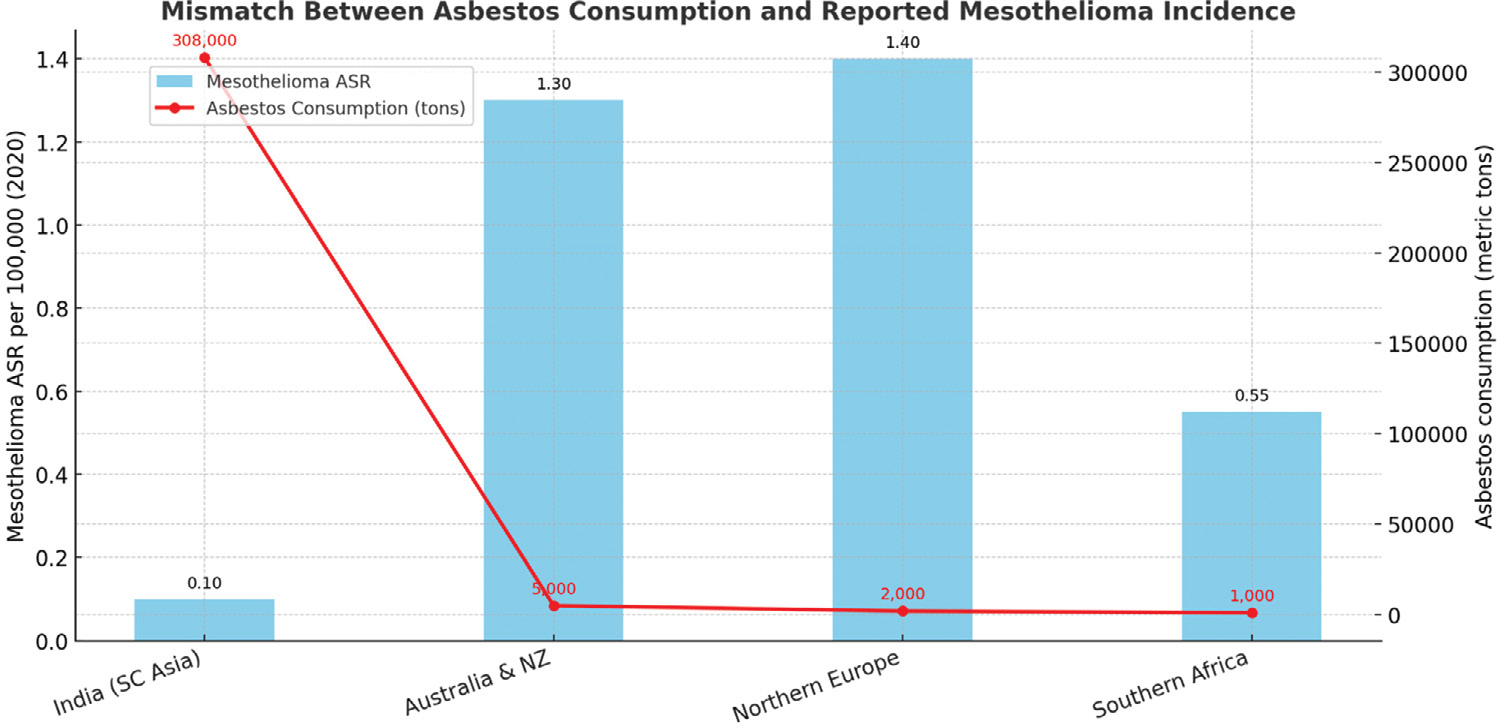
Mismatch between consumption and reported mesothelioma.

1. **Systemic Barriers**
  The unique epidemiological characteristics of ARDs in developing countries present significant challenges in diagnosis and management. Factors such as underreporting, inadequate data collection, limited access to advanced diagnostic tools, and insufficient medical expertise contribute to these difficulties. Underreporting is one of the most pervasive barriers to understanding the true burden of ARDs in LMICs. Underreporting occurs due to a lack of awareness, incomplete investigations, and inadequate registration and documentation of diagnosed cases. Although mandatory health screenings for asbestos-exposed workers are legally mandated, reporting of these notifiable diseases remains largely absent in India. The lack of well-maintained health records, irregular monitoring of workplace asbestos exposure, and high permissible exposure limits further exacerbate the issue. Additionally, many deaths in underdeveloped nations lack a clearly defined cause, making it difficult to determine the true prevalence of asbestos-related lung diseases [20]. Although asbestosis is recognized as a notifiable disease under The Factories Act, 1948, The Mines Act, 1952, and international frameworks such as ILO Convention No. 162, enforcement remains weak in countries like India, whereas developed nations have effectively managed ARDs through stricter occupational safety regulations and advanced healthcare systems [19, 21]. While progress has been made, significant gaps persist in fully understanding the burden of ARDs in developing countries, highlighting the need for stronger regulatory enforcement and improved healthcare infrastructure.
2. **Diagnostic Resource Limitations**
  Diagnosing lung diseases in individuals with a history of asbestos exposure requires a comprehensive assessment, including a detailed occupational history and evaluation of tobacco smoking habits (Figure 2). For suspected malignancies, clinical findings must be correlated with appropriate imaging studies, pulmonary function tests (PFTs), and, when indicated, histological or cytological analysis. A major challenge in diagnosis is the long latency period between initial exposure and disease manifestation. Comparative research involving 23 individuals assessed the diagnostic utility of oblique chest radiographs versus high-resolution computed tomography (HRCT), revealing that circumscribed pleural plaques (CPP) are often incidentally detected in previously exposed patients, particularly on conventional chest X-rays (CXRs) or CT scans. Notably, low-dose (LDCT) demonstrated superior sensitivity in identifying such pleural abnormalities [18]. Unless mesothelioma is suspected, biopsy of pleural plaques is rarely indicated. Another finding is pleural thickening, which is seen due to other causes as well. It is usually distinguished from CPP on plain CXR or CT (computed tomography) imaging. DPT may be seen as round (rolled) atelectasis and parenchymal bands or “crow’s feet” on X-ray imaging.
  According to studies conducted, early parenchymal and pleural changes of asbestosis are more sensitively detected by high-resolution CT scan. Characteristic CT findings are indistinguishable from those of usual interstitial pneumonitis (UIP) and therefore idiopathic pulmonary fibrosis (IPF) [22]. This is why a good history of prior exposure is important.
  The efficacy of LDCT in screening for asbestos-related disorders has increased, with recent studies showing that subtle changes consistent with asbestosis can be reliably described on ultra-low dose CT (ULDCT) [23]. A study was done where two thoracic radiologists independently categorized prone ULDCT scans from 143 participants for interstitial lung disease (ILD) appearances as absent (score 0), probable (1), or definite (2) without knowledge of asbestos exposure or lung function. The results showed probable or definite ILD in 63 (44.1%) participants, concluding that in asbestos-exposed populations, ULDCT may be adequate to detect radiological changes consistent with asbestosis.
  Another study was conducted on 38 male subjects with asbestosis whose plain chest radiographs were read according to the ILO classification independently by 3 observers [24]. The results showed that the measurement of gas transfer (DLCO) is the most sensitive lung function test for asbestosis, and can be abnormal even before the disease is visible on standard radiology [15].
  Typically, asbestosis is diagnosed based on an occupational exposure history and radiologically. There is no need for a lung biopsy as, apart from the presence of asbestos bodies, there are only a few differences between asbestosis and IPF on histology. In a study conducted by Ohar et al. on 3383 asbestos-exposed workers, abnormalities detected on chest radiographs were interpreted by a certified B-reader and were quantified according to the ILO scoring system. Along with spirometry, lung volume measurements and a self-administered questionnaire were also performed [25]. A majority of subjects had either low ILO scores or pleural plaques with no parenchymal abnormalities. Smokers were shown to be younger, had a shorter latency, and a greater ILO score when compared to nonsmokers. These results were consistent with previous studies showing that among asbestos-exposed workers, cigarette smoking is associated with a greater prevalence of parenchymal opacities consistent with asbestosis as smoking paralyzes mucociliary movement. On classifying the subjects according to disease state, only those with mesothelioma showed a restrictive pattern of pulmonary function abnormality [26]. The main finding in participants with both high and low ILO scores, as well as those with bronchogenic malignancy, was an obstructive pattern. This suggests that asbestos-induced lung disease is characterized by low ILO scores, long latencies, greater disease magnitude in smokers, and a normal or obstructive pattern of pulmonary function abnormality.
  Kishimoto and colleagues conducted a study on 152 asbestos-related lung cancer patients in Japan and found that 96% were male, with a median age of 72 years, and 89% were smokers or ex-smokers. The median asbestos exposure duration was 31 years, with a 47-year latency period. Asbestosis was present in 34% of cases, and 81% had pleural plaques, while 62% of operated or autopsied patients had over 5,000 asbestos particles per gram of lung tissue. Asbestosis (34%), pleural plaques with over 10 years of exposure (62%), or asbestos particle concentration (4%) were diagnosed [27].
  Diagnosing ARDs remains a challenge in a large fraction of the exposed population, especially in developing countries. However, other than the resource limitations, poor monitoring, and legislative inadequacies, there are various other diagnostic challenges that play a role too. According to existing literatures, asbestosis has a restrictive pattern, as reported with the occurrence of asbestosis among workers in the United States [28–33]. However, many of these studies had significant limitations. We found that most subjects had normal pulmonary function. In cases where pulmonary function abnormalities were noted, airway obstruction was found to be the dominant finding and occurred prior to the development of restriction. Another finding was spirometric evaluation done in the absence of lung volume measurements which caused misclassification of patients having obstruction with coexistent air trapping as part of the mixed pattern group. Because the lower forced vital capacity (FVC) was attributed to obstruction mixed with restriction rather than obstruction with air trapping, this misinterpretation led to an overestimation of the existence of a restrictive pattern of pulmonary function [25]. In this study, for whichever cases the pulmonologist could not confirm the presence of another disease to which the specific radiological changes, along with exposure to asbestos fibers, could be attributed, it was labeled as pleural disease or lung asbestosis. Further, the results of the study showed characteristic radiological changes of non-malignant asbestos-related pleural disease or lung asbestosis in 14% patients, of whom 22.6% developed malignant pleural mesothelioma (MPM).
  Since pleural plaques and MPM share an overlap in their presentation, a study was performed by focusing to define the appearance of pleural soft tissue masses that could no longer be safely interpreted as typical pleural plaques and could be indicative of an early mesothelioma [34]. For the patients who acquired MPM after their initial screening scan, the study was not able to attribute a particular set of CT changes that were predictive of MPM. It also showed that screening did not lead to the early diagnosis of mesothelioma in any cases. Even though the presence of pleural plaques is not a highly significant risk factor for bronchogenic cancer or malignant mesothelioma (MM), with the increasing popularity in the use of CT for routine diagnostic purposes, the number of individuals being identified with pleural plaques from minor asbestos exposure is increasing. In turn, this leads to raised concerns about the risks of more serious ARDs developing, and resulting in increased diagnostic tests being performed [35]. The inability to reduce MM risk following exposure or to halt the progression of established asbestosis results in significant healthcare problems and expenditure [36].
  Another diagnostic problem with asbestosis is that it cannot be distinguished on clinical or pathological grounds from diffuse interstitial pulmonary fibrosis of other or unknown cause, except on the basis of asbestos exposure evidence (based on history, radiology, or pathology) [37]. History is still the most reliable source of information. In differentiating malignant from BAPE, MRI has been shown to be more sensitive than CT. Histological diagnosis is still key in diagnosis as MRI has a limited role in routine evaluation of malignant pleural disease [38]. 18-Flourodeoxyglucose (FDG) PET can help distinguish between malignant and benign disease, which has a high negative predictive value, but it must also be remembered that false positives may occur in specific cases.
  A histological diagnosis is crucial for planning management and has prognostic significance in MPM based on the cell type found. Cytology may reveal a diagnosis in 33–84% of cases [39]. Distinguishing between malignant mesothelial cells and adenocarcinoma cells on histology can be problematic, thus demonstrating the importance of immunohistochemistry.
  Accurate diagnosis of ARDs often requires access to advanced imaging and pathology, but such resources remain scarce across many LMICs. While chest radiography is inexpensive and widely available, its low sensitivity and specificity limit its ability to detect early disease. More advanced modalities—such as HRCT, LDCT, PET, and MRI—are generally restricted to large urban hospitals (Table 3). Even where such equipment exists, cost barriers and limited referral pathways mean that most asbestos-exposed workers never undergo appropriate imaging.
  Histopathology and immunohistochemistry represent another critical gap. Confirming mesothelioma and distinguishing it from other thoracic malignancies requires specialized pathology services and trained personnel, both of which are concentrated in a handful of academic centers. In many LMICs, suspected cases are either misclassified as generic lung cancer or left unconfirmed due to the absence of diagnostic panels. This not only delays individual patient care but also systematically erases mesothelioma from national statistics.
  The long latency period of ARDs further complicates detection. Workers exposed decades earlier may present only when disease is advanced, by which time diagnostic tools are either unavailable or prohibitively costly. By contrast, high-income countries have integrated HRCT screening, lung function monitoring, and pathology confirmation into structured occupational health programs, making early recognition feasible. The lack of comparable resources in LMICs perpetuates diagnostic delays, underestimation of disease burden, and missed opportunities for prevention and compensation.
3. **Human Resource and Training Gaps**
  Even where diagnostic tools are available, the absence of adequately trained personnel remains a significant barrier in LMICs. Most physicians receive little formal training in occupational medicine, and taking a detailed occupational history is rarely part of routine clinical practice [40, 41]. As a result, many patients with asbestos exposure are treated for their presenting symptoms—such as chronic cough or breathlessness—without any consideration of underlying occupational causes.
  Specialized diagnostic programs that focus on ARDs are almost nonexistent outside a handful of referral centers. In contrast, high-income countries such as the United States and the United Kingdom have developed structured training pathways, occupational lung disease clinics, and registries that enable accurate and early recognition of asbestos-related pathology. This infrastructure allows frontline clinicians to flag high-risk patients, refer them for appropriate imaging or pathology, and capture their diagnoses within surveillance systems.
  In LMICs, the lack of such infrastructure results in a cycle of missed diagnoses and lost data (Figure 3). Without trained occupational health specialists, general practitioners often fail to link clinical findings to asbestos exposure, while pathologists may not have experience with mesothelioma-specific immunohistochemistry panels. The shortage of expertise not only undermines patient care but also prevents the development of accurate national estimates of disease burden, perpetuating the false perception that ARDs are less common in these regions.
4. **Misdiagnosis and Confounding**
  Another critical challenge in LMICs is the frequent misdiagnosis of ARDs, which often mimic other, more common respiratory conditions. Pleural thickening, interstitial fibrosis, or restrictive lung patterns caused by asbestos exposure are easily mistaken for tuberculosis (TB), IPF, or smoking-related chronic lung disease [42]. This diagnostic confusion is particularly acute in regions with high burdens of infectious disease, such as TB, where clinicians may reflexively assign asbestos-exposed workers to a TB treatment regimen without confirmatory testing [43].
  PFTs add to this complexity. ARDs often cause restrictive patterns, but in practice these are frequently misclassified as obstructive changes—especially when smoking is also present. Without the use of high-resolution CT or pathology confirmation, distinguishing between overlapping conditions becomes nearly impossible in resource-limited settings. Consequently, many cases of asbestosis or asbestos-related pleural disease are either missed entirely or subsumed under more generic diagnostic labels [42]. By contrast, in high-income countries, structured diagnostic protocols and greater availability of advanced imaging reduce the likelihood of misclassification. In LMICs, however, the persistence of confounding conditions, combined with limited diagnostic expertise, ensures that many asbestos-related cases remain hidden. This not only undermines patient care but also erodes the accuracy of epidemiological data, reinforcing the cycle of underestimation.
5. **Emerging Technologies and Feasibility**
  In recent years, several emerging technologies have been proposed to improve the early detection of ARDs. These include biomarker-based approaches such as breath analysis, serum assays, and gas chromatography–mass spectrometry (GC-MS) as well as novel imaging modalities like digital tomosynthesis (DTS) and point-of-care ultrasonography (Table 4) [44–47]. While these tools hold considerable promise in high-income countries, their feasibility in LMICs remains limited by cost, infrastructure, and training requirements. Breath-based biomarkers, particularly volatile organic compound (VOC) analysis, are emerging as promising non-invasive tools for ARD detection. Pilot studies using GC-MS, electronic nose (eNose), and proton transfer reaction–mass spectrometry (PTR-MS) have demonstrated encouraging diagnostic accuracy. However, their feasibility in LMICs remains limited due to high costs, technical complexity, and the need for advanced laboratory facilities, cold chain logistics, and highly trained personnel—resources generally scarce outside tertiary centers. Similarly, DTS and LDCT provide improved sensitivity for early asbestos-related changes, but adoption is constrained by high capital investment, limited maintenance capacity, and the uneven distribution of imaging infrastructure. A concise overview of emerging technologies is provided in Table 3, with a detailed comparison of specific VOC platforms available in Supplementary Table S1. By contrast, ultrasonography has emerged as a potentially cost-effective option for detecting pleural abnormalities, but its effectiveness is heavily dependent on operator expertise, which is often limited in resource-constrained health systems (Table 4) [48]. Ultimately, while emerging technologies may offer new pathways for earlier and more accurate diagnosis, their immediate impact in LMICs is likely to be modest without parallel investments in training, infrastructure, and health system strengthening. Critical evaluation of cost-effectiveness, scalability, and portability will be essential to determine which innovations can realistically bridge the diagnostic gap in low-resource settings.

**Table 3:** Established diagnostic modalities for asbestos-related diseases: strengths, limitations, and LMIC applicability.

MODALITY	PROS	CONS	ROLE IN LMICs
**Imaging**			
CXR	Inexpensive, widely available; detects advanced plaques	Low sensitivity; misses early disease; superseded by HRCT	Widely available, poor for early detection
HRCT/LDCT	High sensitivity to early disease; detects small nodules	Costly; radiation exposure; not specific vs. IPF	Limited to tertiary lefts
PET (FDG-PET)	Differentiates benign vs malignant; staging	False positives post-pleurodesis; costly; rare availability	Rare outside research lefts
MRI	Sensitive for pleural malignancy	Expensive; adjunctive only	Very limited use
Ultrasound (US)	Sensitive for small effusions; safe, low-cost	Operator dependent; limited for central lesions	Feasible low-cost adjunct
Digital Tomosynthesis	More sensitive than CXR	Expensive; limited availability	Not yet adopted
**Functional Tests**			
Lung Function (PFT/DLCO)	Non-invasive; DLCO sensitive for early changes	Misclassification risk without lung volume measures	Feasible, underutilized
**Invasive**			
Thoracoscopy	Helps exclude malignancy in BAPE	Invasive, expensive	Limited to specialized lefts
Pleural/Lung Biopsy	Gold standard; essential for compensation	Invasive, risky; requires hospitalization	Rarely accessible, often delayed
Bronchoscopy	Identifies inflammation, obtains tissue	Low sensitivity; invasive	Limited diagnostic value

**Table 4:** Emerging diagnostic technologies: performance, feasibility, and LMIC applicability.

TECHNOLOGY	ACCURACY (VS HRCT/ PATHOLOGY)	COST	PORTABILITY	TRAINING REQUIRED	FEASIBILITY IN LMICs
Breath Biomarkers*	High (pilot studies; VOC-based approaches such as GC-MS, eNose, PTR-MS)	$$$	Portable	High	Low (early stage)
Digital Tomosynthesis	Moderate	$$	Moderate	Moderate	Medium (promising)
Ultrasound	Moderate	$	Highly portable	Moderate	High (low-cost option)

^*^ For detailed comparison of specific VOC platforms, see Supplementary Table S1.

## Recommendation and Future Modalities

Recent research has highlighted several promising modalities for improving the diagnosis of ARDs. Chest ultrasonography has shown potential as a complementary tool to CT scans, demonstrating high sensitivity and specificity in detecting pleural thickening, peripheral lung consolidation, and interstitial changes. A multidisciplinary team (MDT) approach has been proposed for diagnosing BAPE, aiming to standardize criteria and optimize patient management. LDCT has proven significantly more effective than (CXRs) in detecting small non-calcified nodules and early-stage lung cancer in asbestos-exposed individuals. Additionally, non-invasive breath analysis techniques, such as examining exhaled nitric oxide, breath condensate, and VOCs, show promise in identifying biomarkers for asbestosis and mesothelioma. GC-MS has demonstrated particularly high accuracy in differentiating mesothelioma patients from at-risk asbestos-exposed subjects. These emerging diagnostic tools offer the potential for earlier detection, reduced radiation exposure, and improved management of ARDs.

**Figure 4:**
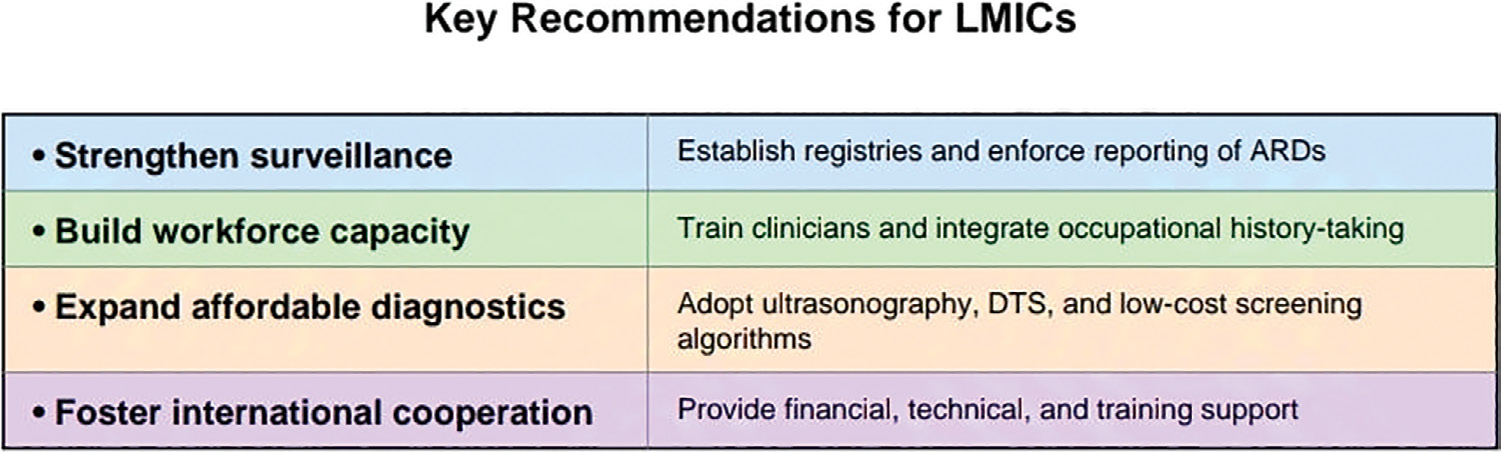
Key recommendation for LMICs.

According to Brusselmans et al., the following criteria, as mentioned in Table 3, are an overview of the benefits and limitations of the different breath-analyzing techniques for VOCs [49]. These innovative, non-invasive diagnostic techniques demonstrate significant potential for detecting asbestos-related malignancies at an early stage, potentially leading to more effective treatment outcomes. Recent research indicates that chest DTS outperforms traditional radiography in both accuracy and sensitivity for identifying pleural plaques. Additionally, DTS exhibits superior sensitivity compared to radiography in diagnosing asbestosis. There is a concerning lack of awareness regarding proper asbestos handling and disposal practices across all levels of organization, from management to workers. Furthermore, occupational health physicians often lack sufficient training to diagnose ARDs due to the absence of dedicated training programs in many countries, unlike the United States or the United Kingdom. Basically, all physicians, everywhere, should be better trained in taking occupational histories (Figure 4).

## Conclusion

With millions of people exposed to asbestos at work and in their homes, ARDs represent a serious global health risk. Asbestos usage persists in many developing nations despite the known health concerns associated with exposure, which contributes to an increase in ARDs in these areas. A number of challenges must be overcome in order to properly diagnose ARDs, such as a shortage of advanced diagnostic instruments, restricted access to healthcare resources, and a lack of knowledge among medical practitioners. Since ARDs can cause severe morbidity and mortality, accurate diagnosis and prompt treatment are still essential. Prevention would be an even better development.

It is essential to put in place thorough policies and standards for the prevention, diagnosis, and treatment of ARDs in order to address these issues. This involves increasing awareness among medical professionals, facilitating better access to diagnostic resources, and creating uniform guidelines for the identification and treatment of ARDs.

Furthermore, assistance from other countries is required to help developing nations put these regulations and standards into practice. To enhance healthcare services and infrastructure, this involves offering financial help, training initiatives, and technical support. By doing these actions, we may contribute to lowering the incidence of ARDs and enhancing the health of asbestos-exposed people everywhere.

## Additional Files

The additional files for this article can be found as follows:

Supplementary Table S1. Benefits and limitations of the different breath-analyzing techniques for VOCs.

Supplementary Figure F1. Mesothelioma incidence (ASR per 100,000) by Region, 2020.

## References

[r1] Frank AL, Joshi TK. The global spread of asbestos. Ann Glob Health. 2014;80(4):257–262. doi:10.1016/j.aogh.2014.09.016. 25459326

[r2] International Agency for Research on Cancer. Asbestos Chrysotile, Amosite, Crocidolite, Tremolite, Actinolite, and Anthophyllite. IARC Monographs on the Evaluation of Carcinogenic Risks to Humans; 2012.

[r3] Mossman BT. Assessment of the pathogenic potential of asbestiform vs. nonasbestiform particulates (cleavage fragments) in vitro (cell or organ culture) models and bioassays. Regul Toxicol Pharmacol. 2008;52(Suppl):S200–S203. doi:10.1016/j.yrtph.2007.10.004. 18006197 PMC2639657

[r4] The Lancet. Asbestos-related disease—a preventable burden. Lancet. 2008;372(9654):1927. doi:10.1016/S0140-6736(08)61821-8. 19059033

[r5] Sokolove Law. Anniversary of U.S. Asbestos Ban: 35 Years Later, Americans Still Lack Protections. Accessed July 11, 2025. https://www.sokolovelaw.com/blog/1989-usa-asbestos-ban-lifted-1991/.

[r6] Wickramatillake BA, Fernando MA, Frank AL. Prevalence of asbestos-related disease among workers in Sri Lanka. Ann Glob Health. 2019;85(1):108. doi:10.5334/aogh.2575. 31322844 PMC6640253

[r7] International Ban Asbestos Secretariat. Asbestos Profile: India. Accessed August 20, 2025. https://ibasecretariat.org/prof_india.php.

[r8] International Labour Organization. Convention C162 - Asbestos Convention, 1986 (No. 162). Accessed February 15, 2025. https://normlex.ilo.org/dyn/nrmlx_en/f?p=NORMLEXPUB:12100:0::NO::P12100_INSTRUMENT_ID:312307.

[r9] Basel Convention. The Basel Convention on the Control of Transboundary Movements of Hazardous Wastes and their. Accessed February 15, 2025. https://www.basel.int/theconvention/overview/tabid/1271/default.aspx. 7697032

[r10] Lin RT, Chien LC, Jimba M, Furuya S, Takahashi K. Implementation of national policies for a total asbestos ban: A global comparison. Lancet Planet Health. 2019;3(8):e341–e348. doi:10.1016/S2542-5196(19)30109-3. 31439315

[r11] Schlünssen V, Mandrioli D, Pega F, et al. The prevalences and levels of occupational exposure to dusts and/or fibres (silica, asbestos and coal): A systematic review and meta-analysis from the WHO/ILO joint estimates of the work-related Burden of disease and injury. Environ Int. 2023;178:107980. doi:10.1016/J.ENVINT.2023.107980. 37487377

[r12] Li N, Zhai Z, Zheng Y, et al. Association of 13 occupational carcinogens in patients with cancer, individually and collectively, 1990–2017. JAMA Netw Open. 2021;4(2):e2037530. doi:10.1001/jamanetworkopen.2020.37530. 33599775 PMC7893501

[r13] LaDou J, Castleman B, Frank A, et al. The case for a global ban on asbestos. Environ Health Perspect. 2010;118(7):897–901. doi:10.1289/ehp.1002285. 20601329 PMC2920906

[r14] American Thoracic Society. Diagnosis and initial management of nonmalignant diseases related to asbestos. Am J Respir Crit Care Med. 2004;170(6):691–715. doi:10.1164/rccm.200310-1436ST. 15355871

[r15] World Health Organization. Chemical Safety and Health. 2024. Accessed February 15, 2025. https://www.who.int/teams/environment-climate-change-and-health/chemical-safety-and-health/health-impacts/chemicals/asbestos.

[r16] Odgerel C, Takahashi K, Sorahan T, et al. Estimation of the global burden of mesothelioma deaths from incomplete national mortality data. Occup Environ Med. 2017;74(12):851–858. doi:10.1136/oemed-2017-104298. 28866609 PMC5740549

[r17] Singh R, Frank AL. Analysis of the Indian government’s position on the use of asbestos and its health effects. Public Health Action. 2023;13(2):50–52. doi:10.5588/pha.23.0013. 37359063 PMC10290263

[r18] Ameille J, Brochard P, Brechot JM, et al. Pleural thickening: A comparison of oblique chest radiographs and high-resolution computed tomography in subjects exposed to low levels of asbestos pollution. Int Arch Occup Environ Health. 1993;64(8):545–548. doi:10.1007/BF00517698. 8314611

[r19] Nishikawa K, Takahashi K, Karjalainen A, et al. Recent mortality from pleural mesothelioma, historical patterns of asbestos use, and adoption of bans: A global assessment. Environ Health Perspect. 2008;116(12):1675–1680. doi:10.1289/ehp.11272. 19079719 PMC2599762

[r20] World Health Organization. WHO Global Plan of Action on Workers’ Health (2008-2017): Baseline for implementation: Global country survey 2008/2009: Executive summary and survey findings. 2013. Accessed Aug 20, 2025. https://www.who.int/publications/i/item/WHO-FWC-PHE-2013-01.

[r21] Singh R, Frank AL. Notification and recordkeeping of occupational mesothelioma in India. MedRxiv. February 14, 2025. doi:10.1101/2025.02.11.25322115. PMC12981211 41652913

[r22] Mathieson JR, Mayo JR, Staples CA, Müller NL. Chronic diffuse infiltrative lung disease: Comparison of diagnostic accuracy of CT and chest radiography. Radiology. 1989;171(1):111–116. doi:10.1148/radiology.171.1.2928513. 2928513

[r23] Manners D, Wong P, Murray C, et al. Correlation of ultra-low dose chest CT findings with physiologic measures of asbestosis. Eur Radiol. 2017;27(8):3485–3490. doi:10.1007/s00330-016-4722-7. 28083692

[r24] Lee YCG, Singh B, Pang SC, de Klerk NH, Hillman DR, Musk AW. Radiographic (ILO) readings predict arterial oxygen desaturation during exercise in subjects with asbestosis. Occup Environ Med. 2003;60(3):201–206. doi:10.1136/oem.60.3.201. 12598668 PMC1740487

[r25] Ohar J, Sterling DA, Bleecker E, Donohue J. Changing patterns in asbestos-induced lung disease. Chest. 2004;125(2):744–753. doi:10.1378/chest.125.2.744. 14769760

[r26] Miller A. Pulmonary function in asbestosis and asbestos-related pleural disease. Environ Res. 1993;61(1):1–18. doi:10.1006/enrs.1993.1044. 8472663

[r27] Baur X. Asbestos-related disorders in Germany: Background, politics, incidence, diagnostics and compensation. Int J Environ Res Public Health. 2018;15(1):143. doi:10.3390/ijerph15010143. 29337930 PMC5800242

[r28] Lerman Y, Seidman H, Gelb S, Miller A, Selikoff IJ. Spirometric abnormalities among asbestos insulation workers. J Occup Med. 1998;30(3):228–233. 3361360

[r29] Brodkin CA, Barnhart S, Anderson G, Checkoway H, Omenn GS, Rosenstock L. Correlation between respiratory symptoms and pulmonary function in asbestos-exposed workers. Am Rev Respir Dis. 1993;148:32–37. doi:10.1164/ajrccm/148.1.32. 8317811

[r30] Wright GW. Functional abnormalities of industrial pulmonary fibrosis. AMA Arch Ind Health. 1955;11:196–203. 14349405

[r31] Selikoff IJ, Churg J, Hammond EC. Asbestos exposure and neoplasia. JAMA. 1964;188(1):22–26. doi:10.1001/jama.1964.03060270028006. 14107207

[r32] Bader ME, Bader RA, Selikoff IJ. Pulmonary function in asbestosis of the lung. Am J Med. 1961;30:235–242. doi:10.1016/0002-9343(61)90095-X. 13685757

[r33] International Labour Organization. 2022 Revised edition of the ILO international classification of radiographs of Pneumoconioses. 2023. Accessed August 21, 2025. https://www.ilo.org/resource/news/2022-revised-edition-ilo-international-classification-radiographs.

[r34] Roberts HC. Screening for malignant pleural mesothelioma and lung cancer in individuals with a history of asbestos exposure. J Thorac Oncol. 2009;4(5):620–628. doi:10.1097/JTO.0b013e31819f2e0e. 19357540

[r35] Reid A, Alfonso H, Ti JSS, Wong E, de Klerk N, Musk AW. Sense of control and wellbeing decades after exposure to blue asbestos at wittenoom, Western Australia. Int J Occup Environ Health. 2012;18:116–123. doi:10.1179/1077352512Z.0000000006. 22762491

[r36] Musk AW, Kler KH, Ambrosini GL, et al. Vitamin A and cancer prevention I: Observations in workers previously exposed to asbestos at Wittenoom, Western Australia. Int J Cancer. 1998;75:355–361. doi:10.1002/(sici)1097-0215(19980130)75:3<355::aid-ijc5>3.0.co;2-1. 9455793

[r37] Henderson DW, Jones ML, Klerk ND, et al. The diagnosis and attribution of asbestos-related diseases in an Australian context: Report of the adelaide workshop on asbestos-related diseases. October 6–7, 2000. Int J Occup Environ Health. 2004;10:40–46. doi:10.1179/oeh.2004.10.1.40. 15070024

[r38] Schuhmann M, Brims F, O’Reilly KMA. Asbestos-related lung disease an update. Clin Pulm Med. 2011;18:265–273. doi:10.1097/CPM.0b013e318235181f.

[r39] Whitaker D. The cytology of malignant mesothelioma. Cytopathology. 2000;11:139–151. doi:10.1046/j.1365-2303.2000.00247.x. 10877273

[r40] Walker-Bone K, Hollick R. Health and work: What physicians need to know. Clin Med (Lond). 2021;21:195–200. doi:10.7861/clinmed.2020-0847. 33947660 PMC8140699

[r41] Alex R, Francis M, Prashanth HR, Kundavaram A. Occupational history: A neglected component of history taking. Indian J Occup Environ Med. 2013;17:29–30. doi:10.4103/0019-5278.116371. 24082646 PMC3777287

[r42] Supernova Rotecting Your World. Can Lung Cancer Caused by Asbestos Exposure be Misdiagnosed or Mistaken for Other Types of Lung Cancer? – Understanding the Risk and Implications. 2023. Accessed August 21, 2025. https://asbestos-surveys.org.uk/asbestos/the-link-between-asbestos-and-lung-cancer/can-lung-cancer-caused-by-asbestos-exposure-be-misdiagnosed-or-mistaken-for-other-types-of-lung-cancer/.

[r43] Roy p, Bardhan M, Roy S, Singh U, Suresh T, Anand A. Silico-tuberculosis amidst COVID-19 pandemic: Global scenario and Indian perspective. Ann Med Surg (Lond). 2023;84:6083–6090. doi:10.1097/MS9.0000000000001471. PMC10718399 38098595

[r44] Lee G, Jeong YJ, Kim KI, et al. Comparison of chest digital tomosynthesis and chest radiography for detection of asbestos-related pleuropulmonary disease. Clin Radiol. 2013;68(4):376–382. doi:10.1016/j.crad.2012.05.022. 23177084

[r45] Lamote K, Brinkman P, Vandermeersch L, et al. Breath analysis by gas chromatography-mass spectrometry and electronic nose to screen for pleural mesothelioma: A cross-sectional case-control study. Oncotarget. 2017;8(53):91593–91602. doi:10.18632/oncotarget.21335. 29207669 PMC5710949

[r46] Smallwood N, Dachsel M. Point-of-care ultrasound (POCUS): Unnecessary gadgetry or evidence-based medicine? Clin Med (Lond). 2018;18:219–224. doi:10.7861/clinmedicine.18-3-219. 29858431 PMC6334078

[r47] Smargiassi A, Pasciuto G, Pedicelli I, et al. Chest ultrasonography in health surveillance of asbestos-related lung diseases. Toxicol Ind Health. 2017;33:537–546. doi:10.1177/0748233716686916. 28162043

[r48] Shah S, Umulisa I, Noble VE, et al. Development of an ultrasound training curriculum in a limited resource international setting: Successes and challenges of ultrasound training in rural Rwanda. Int J Emerg Med. 2008;1:193–196. doi:10.1007/s12245-008-0053-z. 19384515 PMC2657276

[r49] Brusselmans L, Arnouts L, Millevert C, Vandersnickt J, van Meerbeeck JP, Lamote K. Breath analysis as a diagnostic and screening tool for malignant pleural mesothelioma: A systematic review. Transl Lung Cancer Res. 2018;7:520–536. doi:10.21037/tlcr.2018.04.09. 30450290 PMC6204411

