# Supplementary material for: Challenges in Identifying and Diagnosing Asbestos-Related Diseases in Emerging Economies: A Global Health Perspective
Source: Ann Glob Health. 2025 Sep 18;90(1):65. doi: 10.5334/aogh.4871 (PMC12458074; doi:10.5334/aogh.4871)
Supplement: Supplementary Table S1. — Benefits and limitations of the different breath-analyzing techniques for VOCs. [file agh-91-1-4871-s1.pdf]

***Supplementary Table S1*: Benefits and limitations of the different breath analyzing techniques for VOCs**

**Characteristic**	**GC-MS**	**IMS**	**eNose**	**Canines**	**SIFT-MS**	**PTR-MS**
Sensitivity	+++	++	+/-	● -	++	+
Real-time?	No	Yes	No	Yes	Yes	Yes
Sampling	Offline	Online	Offline	Online	Online/offline	Online/offline
User-dependence	Need of qualified technicians	User-friendly	User-friendly	User-friendly	User-friendly, interpretation by specialist	User-friendly, interpretation by specialist
Time	Slow, time-consuming	Fast, easy	Fast, easy	Fast, easy	Fast, easy	Fast, easy
Price	+++	+	+	++	+++	+++
Transportable	No	Yes	Yes	Yes	No	No
VOC Identification?	Yes	Pseudo	No	No	No	No

+, medium; ++, high; +++, very high; −, low; −−, very low. eNose, Electronic nose; GC-MS, gas chromatography-mass spectrometry; IMS, ion mobility spectrometry; MCC, multi-capillary column; PTR-MS, proton transfer reaction-mass spectrometry; SIFT-MS, selected ion flow tube-mass spectrometry; VOC, volatile organic compound
