# Supplementary figures and images for: Challenges in Identifying and Diagnosing Asbestos-Related Diseases in Emerging Economies: A Global Health Perspective
Source: Ann Glob Health. 2025 Sep 18;90(1):65. doi: 10.5334/aogh.4871 (PMC12458074; doi:10.5334/aogh.4871)

**Supplementary Figure F1: Mesothelioma incidence (ASR per 100,000) by Region, 2020.**

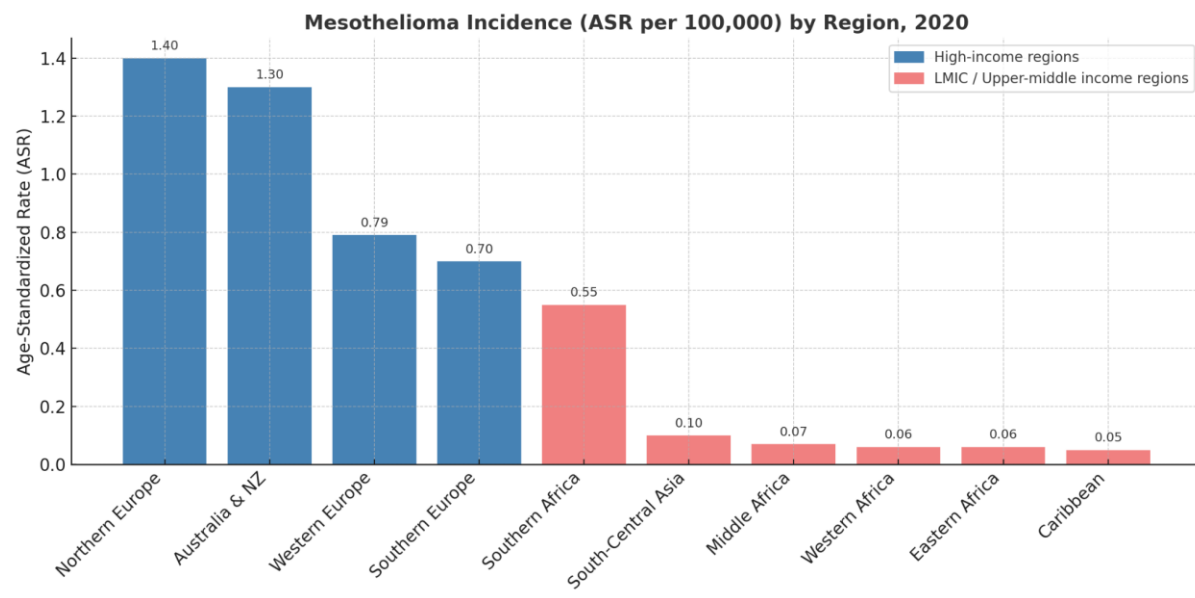

Supplement: Supplementary Figure F1. — Mesothelioma incidence (ASR per 100,000) by Region, 2020. [file agh-91-1-4871-s2.pdf]
